# Supplementary material for: Niche-directed evolution modulates genome architecture in freshwater Planctomycetes
Source: ISME J. 2019 Jan 4;13(4):1056–71. doi: 10.1038/s41396-018-0332-5 (PMC6461901; doi:10.1038/s41396-018-0332-5)
Supplement: Supplementary file 1 — Supplementary materials and methods [file 41396_2018_332_MOESM1_ESM.docx]

Niche-directed evolution modulates genome architecture in freshwater Planctomycetes

Running title: Niche-directed evolution of Planctomycetes genomes

Adrian-Ştefan Andrei^1*^, Michaela M. Salcher^2^, Maliheh Mehrshad^1^, Pavel Rychtecký^1^, Petr Znachor^1^, Rohit Ghai^1*^

^1^*Institute of Hydrobiology, Department of Aquatic Microbial Ecology, Biology Centre of the Academy of Sciences of the Czech Republic, České Budějovice, Czech Republic*.

^2^*Limnological Station, Institute of Plant and Microbial Biology, University of Zurich, strasse 187, CH-8802 Kilchberg, Switzerland*.

*Corresponding authors: Adrian-Ştefan Andrei & Rohit Ghai

Institute of Hydrobiology, Department of Aquatic Microbial Ecology, Biology Centre of the Academy of Sciences of the Czech Republic, Na Sádkách 7, 370 05, České Budějovice, Czech Republic

Phone: +420 387 775 881

Fax: +420 385 310 248

E-mails: adrian.stefan.andrei@hbu.cas.cz; ghai.rohit@gmail.com

Supplementary Information

Materials and methods

Binning

For generating Planctomycetes metagenome-assembled genomes (MAGs), we used a combination of taxonomy-dependent and -independent binning techniques (see Supplementary material). Firstly, a supervised alignment-based method (e.g. taxonomic assignment according to the best hits) was used on the assembled contigs that had a minimum length of 5 kbp. Briefly, their protein coding sequences predicted by MetaProdigal [1] were annotated using UBLAST [2] (with the lenient cut-offs: E-value 1e-3, similarity 10%, coverage 10%, bitscore 50) against an in-house curated Prokaryotic RefSeq Genomes Release 81 database, that was amended by addition of supplementary genomes. The revision of RefSeq database was done by including all Planctomycetes genomes (including MAGs and SAGs) publicly available in NCBI Genome database (i.e. 102 entries in May 2017). The genomes that were affiliated to Planctomycetes phylum (as predicted by PhyloPhlAn) and had a genome completeness (as estimated by CheckM) [3] higher than 10% were merged with the above-mentioned RefSeq release. Prior to inclusion in RefSeq, 13 genomes (that lacked annotation) were annotated using Prokka [4]. Secondly, we selected from all the assembled metagenomic datasets (i.e. 10 datasets) the contigs with a minimum length of 5 kbp that gave more than 50% best UBLAST hits to Planctomycetes (if from the total number of proteins present in one contig more than 50% gave hits to Planctomycetes we considered the contig belonging to Planctomycetes phylum) and used them further for taxonomy–independent binning. For this step, we used the contigs’ mean base coverage, computed by bbwrap.sh (with default parameters) [5], to perform hybrid binning using tetranucleotide frequencies and abundance data *via* MetaBAT [6] (using the presets –superspecific and --minCorr 99). Prior to downstream analyses, the bins that were found to be poorly resolved (i.e. have more than 10% redundancy) were further refined using anvi’o software [7] as described elsewhere (http://merenlab.org/2016/06/22/anvio-tutorial-v2/). The obtained bins that were found to be taxonomically affiliated with Planctomycetes (as established by PhyloPhlAn) and to have a genome completeness higher than 10% (as determined by using 360 Planctomycetes marker genes in CheckM) were denominated as Planctomycetes MAGs.

Planctosome model construction

The annotation of the carbohydrate-active enzymes (using the dbCAN-seq database; [8]) present in the freshwater Planctomycetes MAGs showed the simultaneous occurrence of cohesin and dockerin modules in the genomes of the Nemodlikiaceae family (Supplementary Figure 13). The nature of the high-affinity/stability protein-protein interaction characteristic for the cohesin-dockerin complex and its portrayal as a signature component of the cellulosomal machinery (extracellular multi-enzyme complex utilised by anaerobic bacteria for the degradation of lignocellulosic biomass; [9]) motivated us to look deeper at the proteins encoding cohesin and dockerin domains. In doing so, we used a locally installed version of InterProScan (version 5.24-63.0, default settings and databases) [10] to annotate protein domains. The proteins found to encode cohesion and/or dockerin domains were further scrutinised for the presence of signal peptides and cellular localization using Phobius [11]. This annotation was cross-checked and refined using CDD [12], hmmscan (with Pfam, TIGRFAM, Gene3D, Superfamily, PIRSF and TreeFam databases) [13], jackhmmer [13] and Phyre2 [14]. Subsequently, the homology and structure-based predictions were coupled with protein domain co-occurrence patterns (performed using the Pfam’s domain organization database) and used for functional inferences. For example, we observed that reprolysin, proprotein convertase p-domains and thrombospondin repeats tend to co-occur. The protein domain co-occurences and functional inferences suggested that the cohesin/dockerin containing proteins present in the MAG ZH-13MAY13-plancto74 could interact, likely forming a complex of three proteins which resembles in overall architecture the simple cellulosome systems found in some mesophilic bacteria (e.g. *Ruminococcus bromii*) [9]. For this complex we propose the name “planctosome” and highlight its putative role in polypeptide degradation in Figure 5.

CARD-FISH

The RAxML tree (Supplementary Figure S4) served as backbone, and probes for 10 monophyletic lineages containing MAG sequences or high amounts of sequences extracted from reads were subjected to probe design using the probe_design and probe_check tools in ARB [15]. The resulting probes were checked *in silico* using mathFISH [16] and in the laboratory with different formamide concentrations until stringent hybridization conditions were achieved. CARD-FISH was performed for 28 samples from Římov Reservoir and 45 samples from Lake Zurich collected in 2015. Římov Reservoir was sampled during the spring phytoplankton bloom (April 14^th^), early summer (June 16^th^), late summer (August 10^th^), and autumn (November 04^th^) at 0, 5, 10, 20, 30, and 40m depths and Lake Zurich was sampled during winter mixis (February 4^th^), the spring phytoplankton bloom (April 15^th^), early summer (June 11^th^), late summer (August 11^th^), and autumn (November 3^th^) at 0, 5, 10, 20, 30, 40, 60, 80, and 100m depths. CARD-FISH was done with fluorescein-labeled tyramides as previously described [17] and analyzed by fully automated high-throughput microscopy [18]. Interfering autofluorescent cyanobacteria or debris particle were individually excluded from hybridized cells and at least 10 high quality images or >1000 DAPI stained bacteria were analyzed per sample. Micrographs of CARD-FISH stained Planctomycetes lineages were recorded with a highly sensitive charge-coupled device (CCD) camera (Vosskühler) at a magnification of 1000 x and cell sizes were estimated using the software LUCIA (Laboratory Imaging Prague, Czech Republic) following a previously described workflow [19].

Software versions

hmmsearch version 3.1b2

Kalign version 2.04

FastTree version 2.1.7

MAFFT version 7.055b

USEARCH version 6

BBMap version 36.x

MEGAHIT version 1.1.2

MetaBAT version 0.32.4

reformat.sh version 36.19

uclust version 1.2.22q

ublast version 6

SSU-ALIGN version 0.1.1

blastall version 2.2.21

MetaProdigal version 2.6.3

bbwrap.sh version 36.x

PhyloPhlAn version 1.7

CheckM version 1.0.11

Prokka version 1.11

BlastKOALA version 2.1

barrnap version 0.8

tRNAscan-SE version 1.4

InterProScan version 5.25-64.0

HmmerWeb version 2.26.0

Phyre2 version 2.0

Phobius version 1.01

BMGE version 1.12

MUSCLE version 3.8.31

SINA version 1.2.11

RAxML version 7.2.8

ARB version 6.0.6

LUCIA version 1.0

Database versions

SILVA 16S rRNA database version SSURef_NR99_132 database38

RefSeq 81 database: Downloaded on March 30, 2018

Clusters of Orthologous Groups: http://www.ncbi.nlm.nih.gov/COG/

InterPro databases as available in version InterProScan Version 5.24-63.0: TIGRFAM,SMART,Pfam,Hamap,ProDom,PRINTS,CDD,SUPERFAMILY,Gene3D,PIRSF

dbCAN-seq database version 5

References

1. Hyatt D, Chen GL, LoCascio PF, Land ML, Larimer FW, Hauser LJ. Prodigal: Prokaryotic gene recognition and translation initiation site identification. *BMC Bioinformatics* 2010; **11**.

2. Edgar RC. Search and clustering orders of magnitude faster than BLAST. *Bioinformatics* 2010; **26**: 2460–2461.

3. Parks DH, Imelfort M, Skennerton CT, Hugenholtz P, Tyson GW. CheckM: Assessing the quality of microbial genomes recovered from isolates, single cells, and metagenomes. *Genome Res* 2015; **25**: 1043–1055.

4. Seemann T. Prokka: Rapid prokaryotic genome annotation. *Bioinformatics* 2014; **30**: 2068–2069.

5. Bushnell B. BBWrap. 2015.

6. Kang DD, Froula J, Egan R, Wang Z. MetaBAT, an efficient tool for accurately reconstructing single genomes from complex microbial communities. *PeerJ* 2015; **3**: e1165.

7. Eren AM, Esen ÖC, Quince C, Vineis JH, Morrison HG, Sogin ML, et al. Anvi’o: an advanced analysis and visualization platform for ‘omics data. *PeerJ* 2015; **3**: e1319.

8. Huang L, Zhang H, Wu P, Entwistle S, Li X, Yohe T, et al. DbCAN-seq: A database of carbohydrate-active enzyme (CAZyme) sequence and annotation. *Nucleic Acids Res* 2018; **46**: D516–D521.

9. Artzi L, Bayer EA, Moraïs S. Cellulosomes: Bacterial nanomachines for dismantling plant polysaccharides. *Nat Rev Microbiol* 2017; **15**: 83–95.

10. Jones P, Binns D, Chang H-Y, Fraser M, Li W, McAnulla C, et al. InterProScan 5: genome-scale protein function classification. *Bioinformatics* 2014; **30**: 1236–1240.

11. Käll L, Krogh A, Sonnhammer ELL. Advantages of combined transmembrane topology and signal peptide prediction—the Phobius web server. *Nucleic Acids Res* 2007; **35**: W429–W432.

12. Marchler-Bauer A, Derbyshire MK, Gonzales NR, Lu S, Chitsaz F, Geer LY, et al. CDD: NCBI’s conserved domain database. *Nucleic Acids Res* 2015; **43**: D222–D226.

13. Finn RD, Clements J, Arndt W, Miller BL, Wheeler TJ, Schreiber F, et al. HMMER web server: 2015 Update. *Nucleic Acids Res* 2015; **43**: W30–W38.

14. Kelley LA, Mezulis S, Yates CM, Wass MN, Sternberg MJE. The Phyre2 web portal for protein modeling, prediction and analysis. *Nat Protoc* 2015; **10**: 845.

15. Ludwig W, Strunk O, Westram R, Richter L, Meier H, Yadhukumar A, et al. ARB: A software environment for sequence data. *Nucleic Acids Res* 2004; **32**: 1363–1371.

16. Yilmaz LS, Parnerkar S, Noguera DR. MathFISH, a web tool that uses thermodynamics-based mathematical models for in silico evaluation of oligonucleotide probes for fluorescence in situ hybridization. *Appl Environ Microbiol* 2011; **77**: 1118–1122.

17. Sekar R, Pernthaler A, Pernthaler J, Posch T, Amann RI, Warnecke F. An Improved Protocol for Quantification of Freshwater Actinobacteria by Fluorescence In Situ Hybridization An Improved Protocol for Quantification of Freshwater Actinobacteria by Fluorescence In Situ Hybridization. *Appl Environ Microbiol* 2003; **69**: 2928–2935.

18. Zeder M, Pernthaler J. Multispot live-image autofocusing for high-throughput microscopy of fluorescently stained bacteria. *Cytom Part A* 2009; **75**: 781–788.

19. Posch T, Franzoi J, Prader M, Salcher MM. New image analysis tool to study biomass and morphotypes of three major bacterioplankton groups in an alpine lake. *Aquat Microb Ecol* 2009; **54**: 113–126.
