# Supplementary material for: Niche-directed evolution modulates genome architecture in freshwater Planctomycetes
Source: ISME J. 2019 Jan 4;13(4):1056–71. doi: 10.1038/s41396-018-0332-5 (PMC6461901; doi:10.1038/s41396-018-0332-5)
Supplement: Supplementary file 2 — Supplementary [file 41396_2018_332_MOESM2_ESM.pdf]

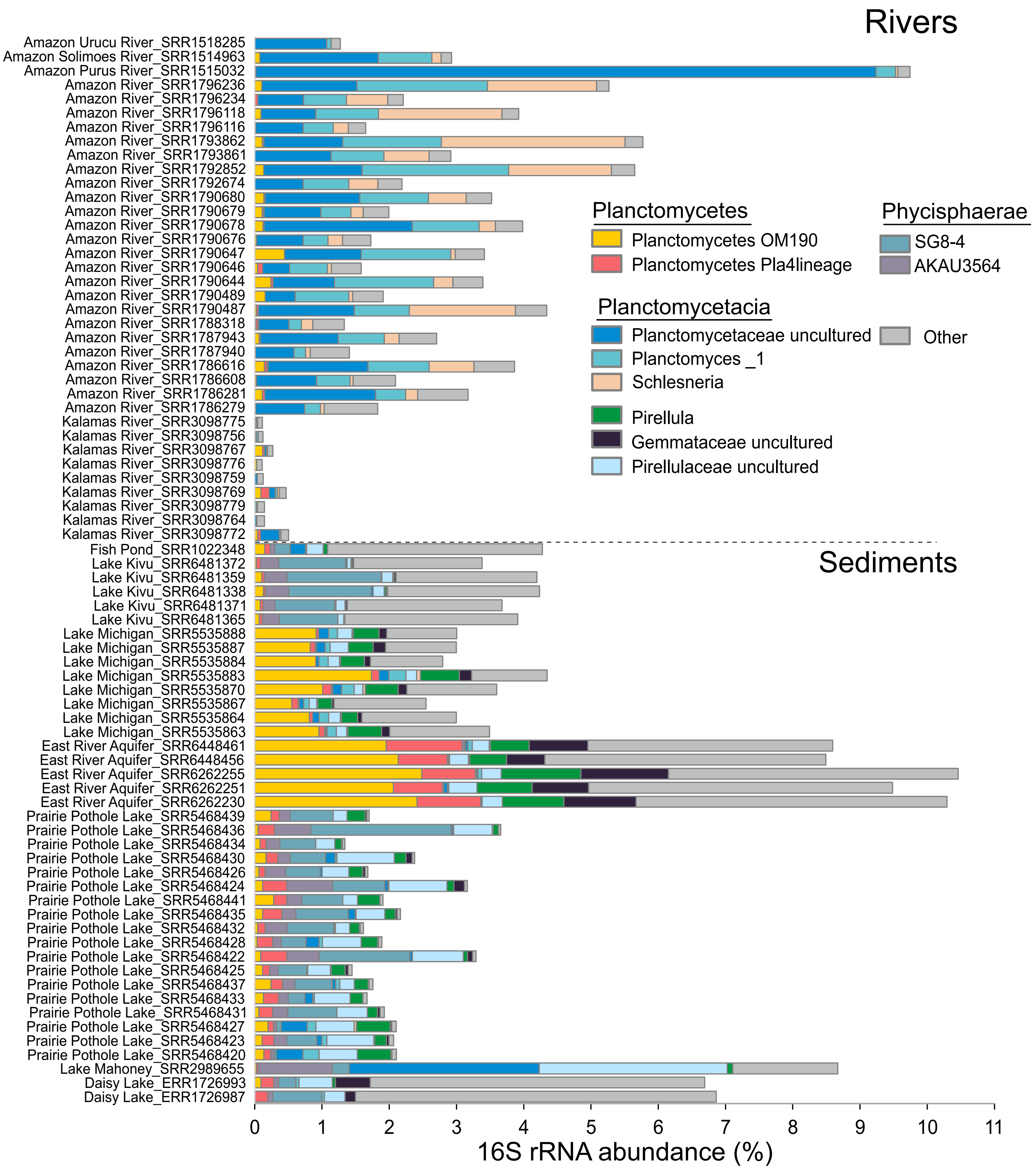

Supplementary Figure 1. Taxonomic profile of Planctomycetes found in riverine and sediments ecosystems. The figure depicts the SILVA SSU (Ref NR 99 128) classification of 16S rRNA gene fragments (as unassembled shotgun reads) retrieved from 76 metagenomic datasets (36 from rivers and 40 from sediments). The X-axis indicates the percentage of Planctomycetes within the prokaryotic communiities (as assessed by 16S rRNA abundance), while the Y-axis shows the collection sites and their respective SRA identifiers.

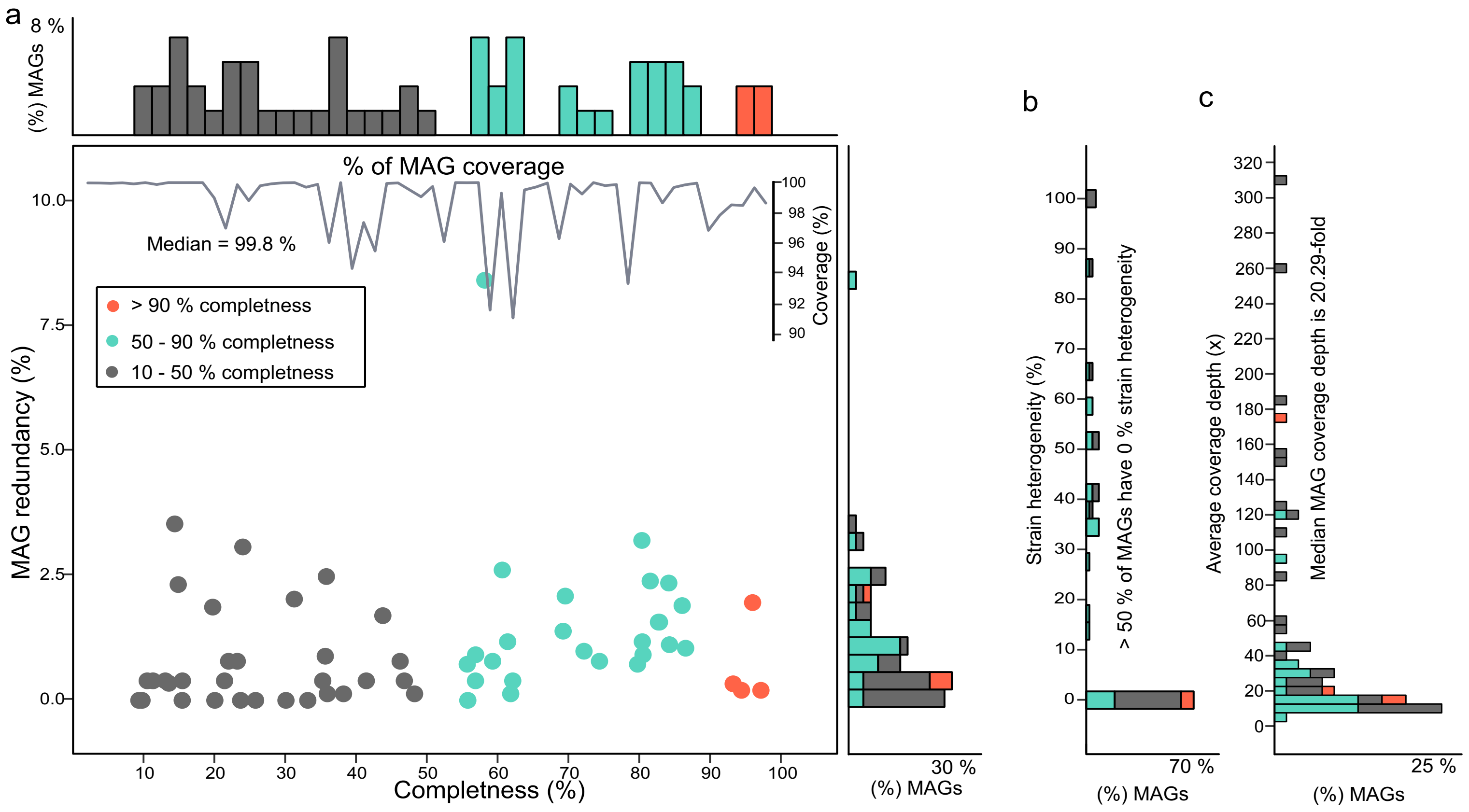

Supplementary Figure 2. Metagenome-assembled genomes (MAGs) quality assessments. The figure shows the levels of completeness, redundancy and genomic coverage for all 60 Planctomycetes MAGs. The histograms situated along the X and Y axes depict the percentages of genomes that have varying levels of completeness and heterogeneity. The panels b and c, show MAG heterogeneity and average coverage depth (from the metagenome of origin).

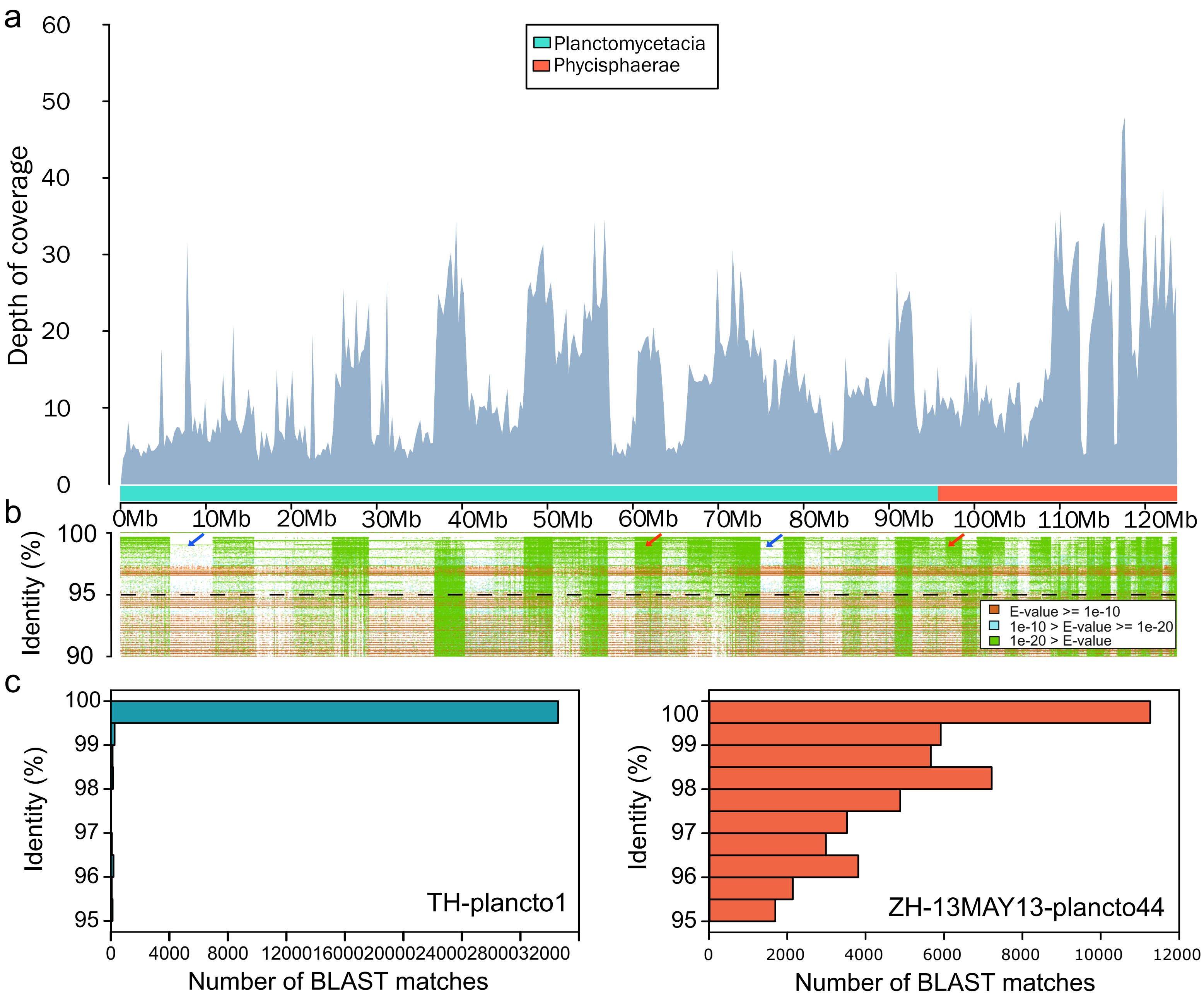

Supplementary Figure 3. Planctomycetes MAGs recruitment plots. The panel a) shows the recruitment pattern of 180 million metagenomic reads (subsampled from the environments from where the MAGs where recovered) against the concatenated genomes of 60 MAGs. The X-axis scale shows the total genomic length, while and the Y-one shows the coverage depth. The coloured bar situated above the X-axis indicates the taxonomic affiliation of the respective DNA fragment (dark cyan for Planctomycetacia, and red for Phycisphaerae). Panel b) shows the alignment identity(%) between metagenomic reads and the MAGs' nucleotide sequences. Individual reads are coloured based on E-value scores (see legend in the left part of the panel). Blue arrows indicate regions with high intra-population diversity, while red arrows point towards 'sequence-discrete' populations.The histograms in panel c) show the number of reads (from 20 million reads subsets) (X-axis) that have identities ≥ 95% with Planctomycetes MAGs. The MAG TH-plancto1 is a representative of 'sequence-discrete' species, while ZH-13MAY13-plancto44 of high diversity one.

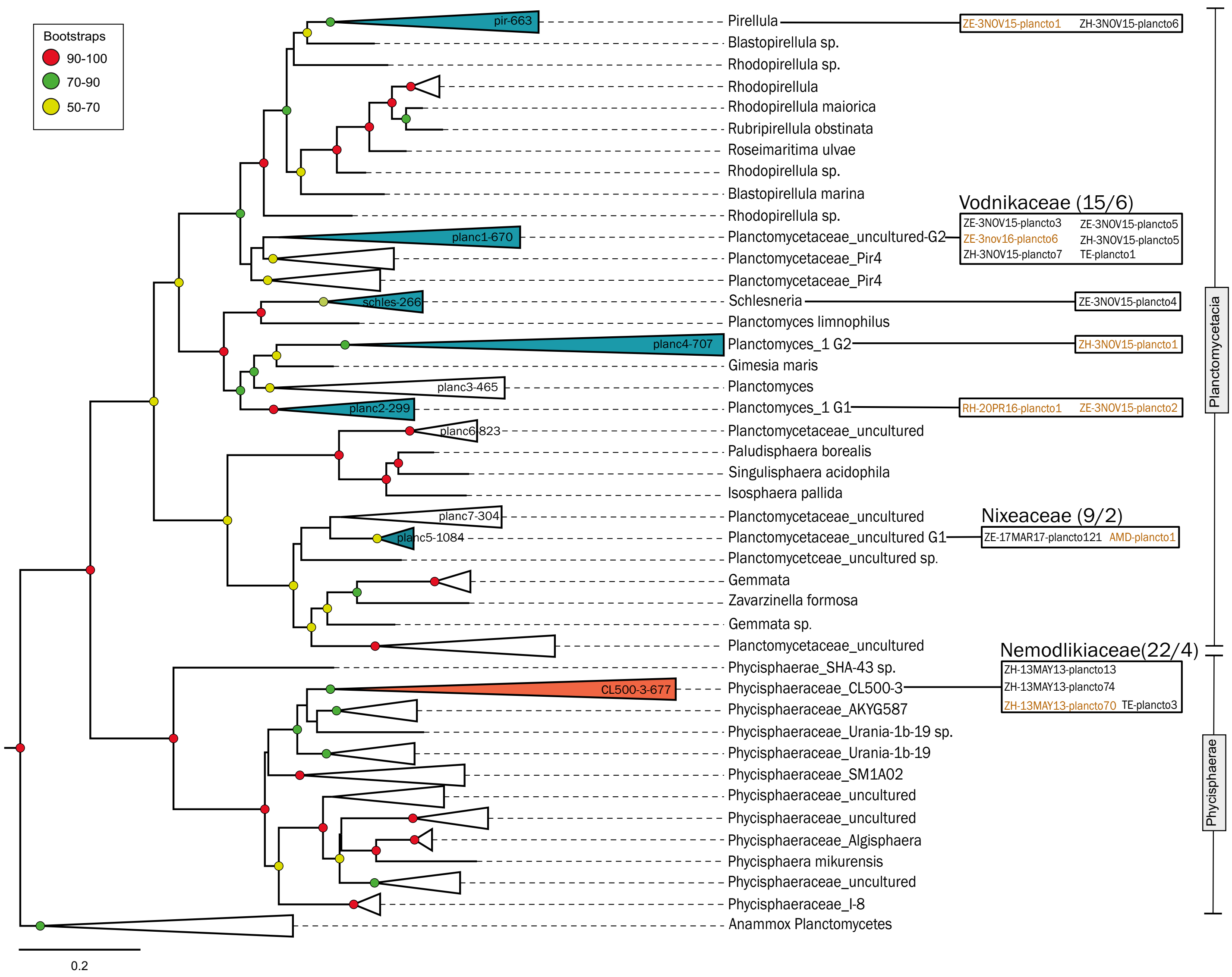

Supplementary Figure 4. Maximum likelihood 16S rRNA phylogenetic trees. The names of the collapsed branches indicate the CARD-FISH probe that targeted the respective group. The left panels indicate the MAGs (from the respective clades) that had 16S rRNA gene sequences. The 16S rRNA gene sequences recovered from the MAGs with red colored names were used for CARD-FISH probe design. The strength of support for internal nodes was assessed by performing 100 bootstrap replicates, with the obtained values shown as colored circles (top left legend).

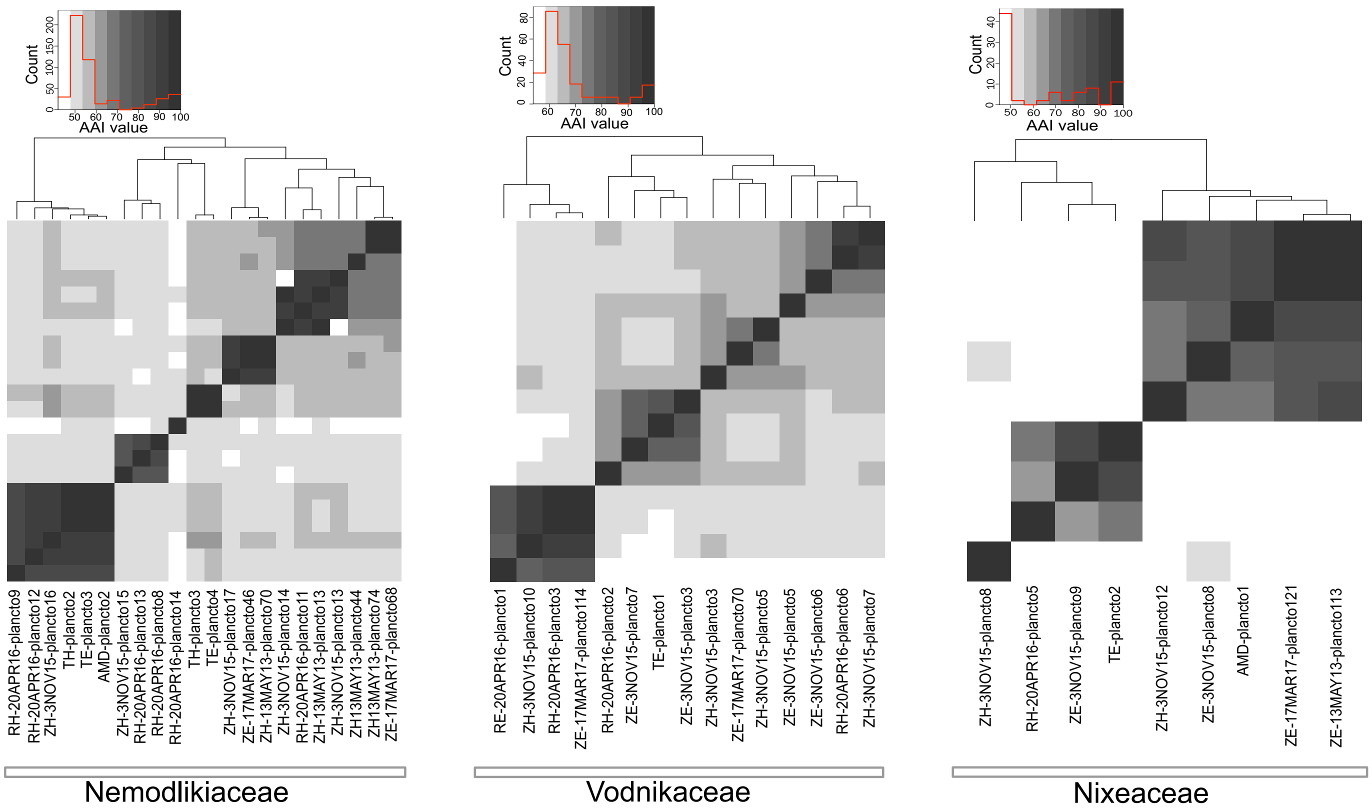

Supplementary Figure 5. Heat maps of average amino acid identities between the MAGs belonging to Nemodlikiaceae, Nixeaceae and Vodnikaceae families. The dendrograms positioned above the graphs show hierarchical clustering relationships between related MAGs. The names of the MAGs and the phylogenomic group they belong to are shown under each heat map. The upper panels display the color key histograms.

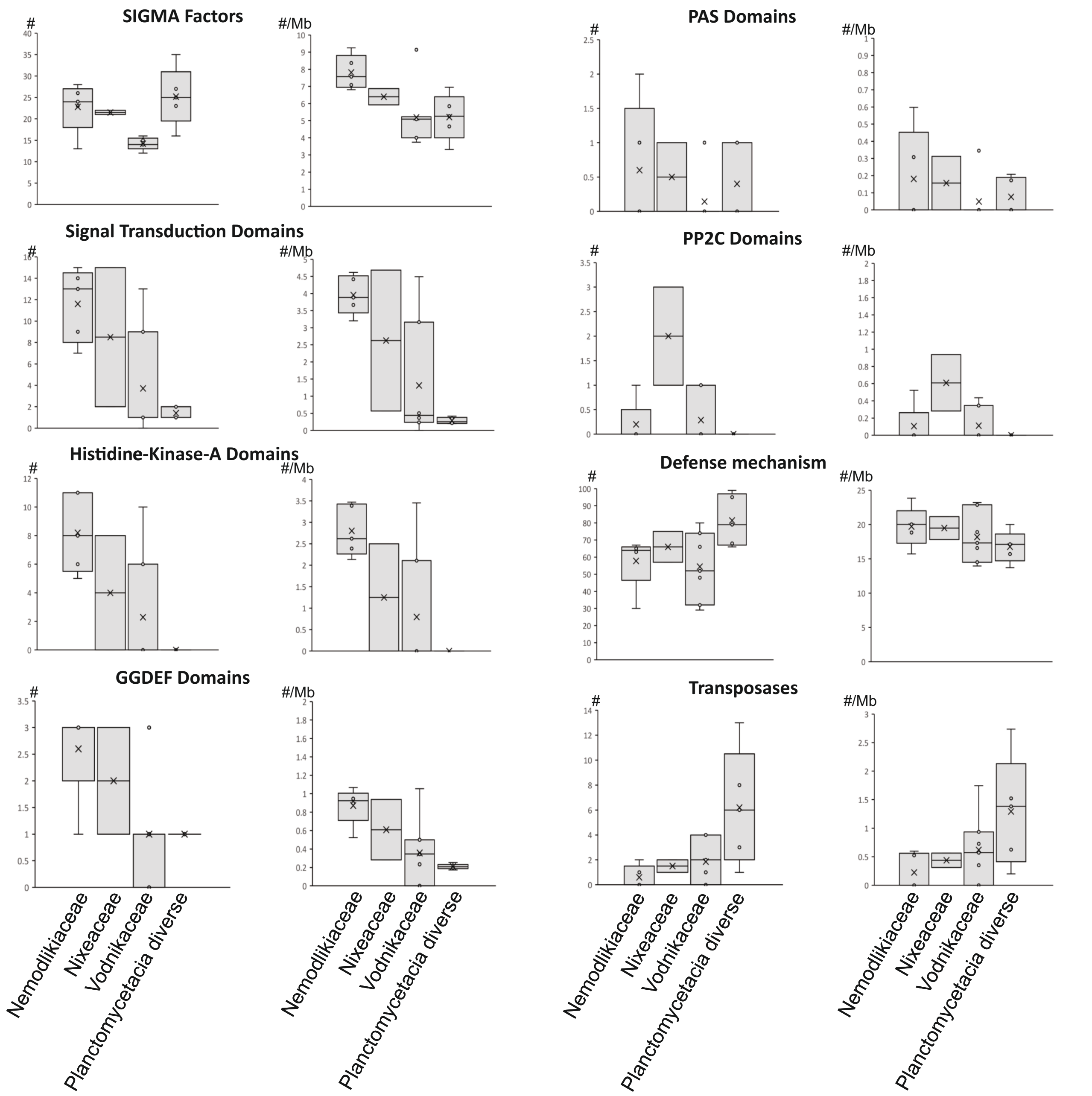

Supplementary Figure 6. The distribution of different protein domains and functional categories across different Planctomycetes groups. Both total numbers found and the total number/Mb are shown for all groups.

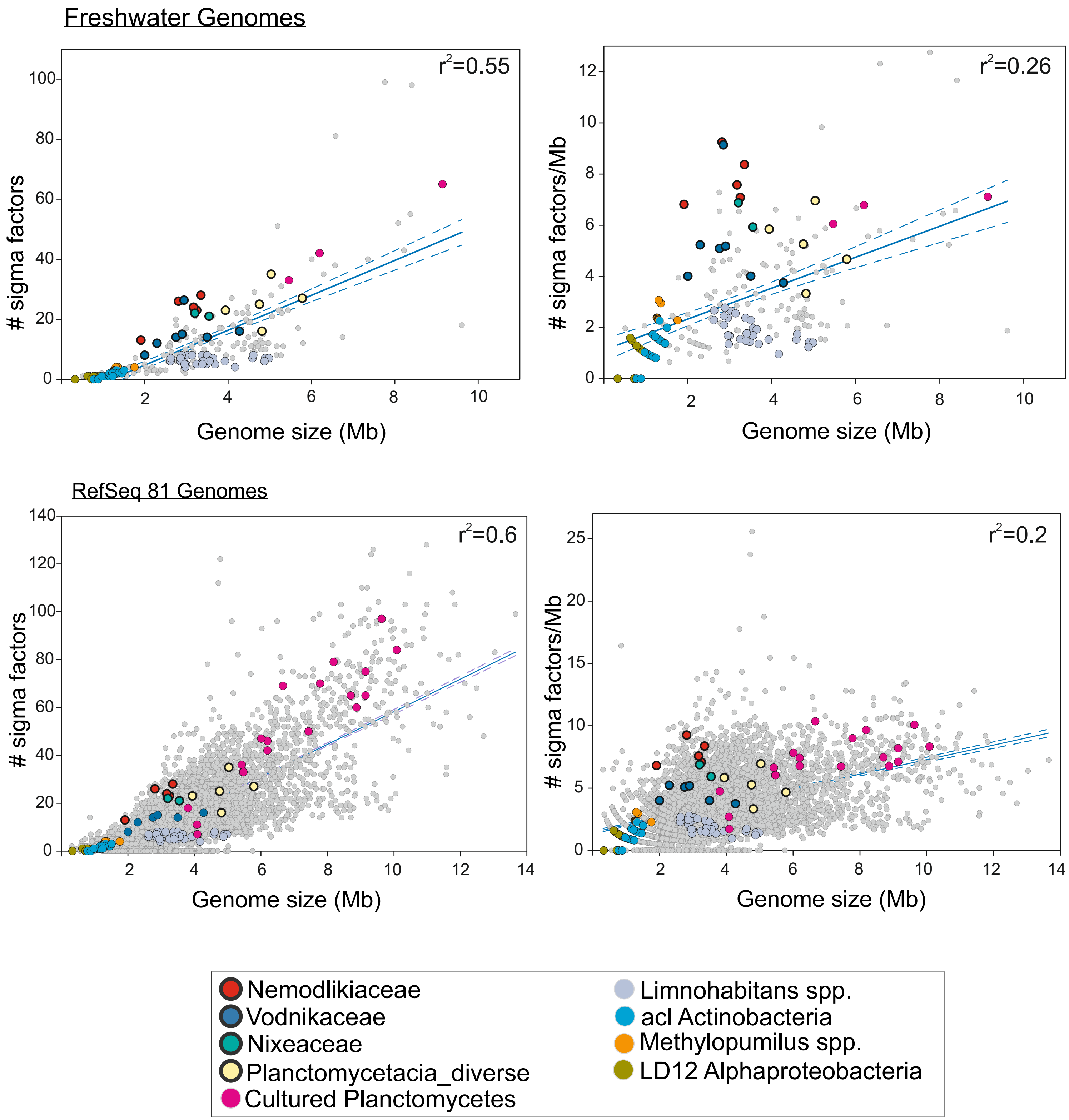

Supplementary Figure 7: Sigma Factors in Freshwater Genomes (Top) and RefSeq Release 81 Genomes (Bottom). Both the total number of sigma factors found and the total number per Mb are shown as a function of Genome Size (Mb). Planctomycetes genomes along with some representative freshwater microbes are also shown in different colors.

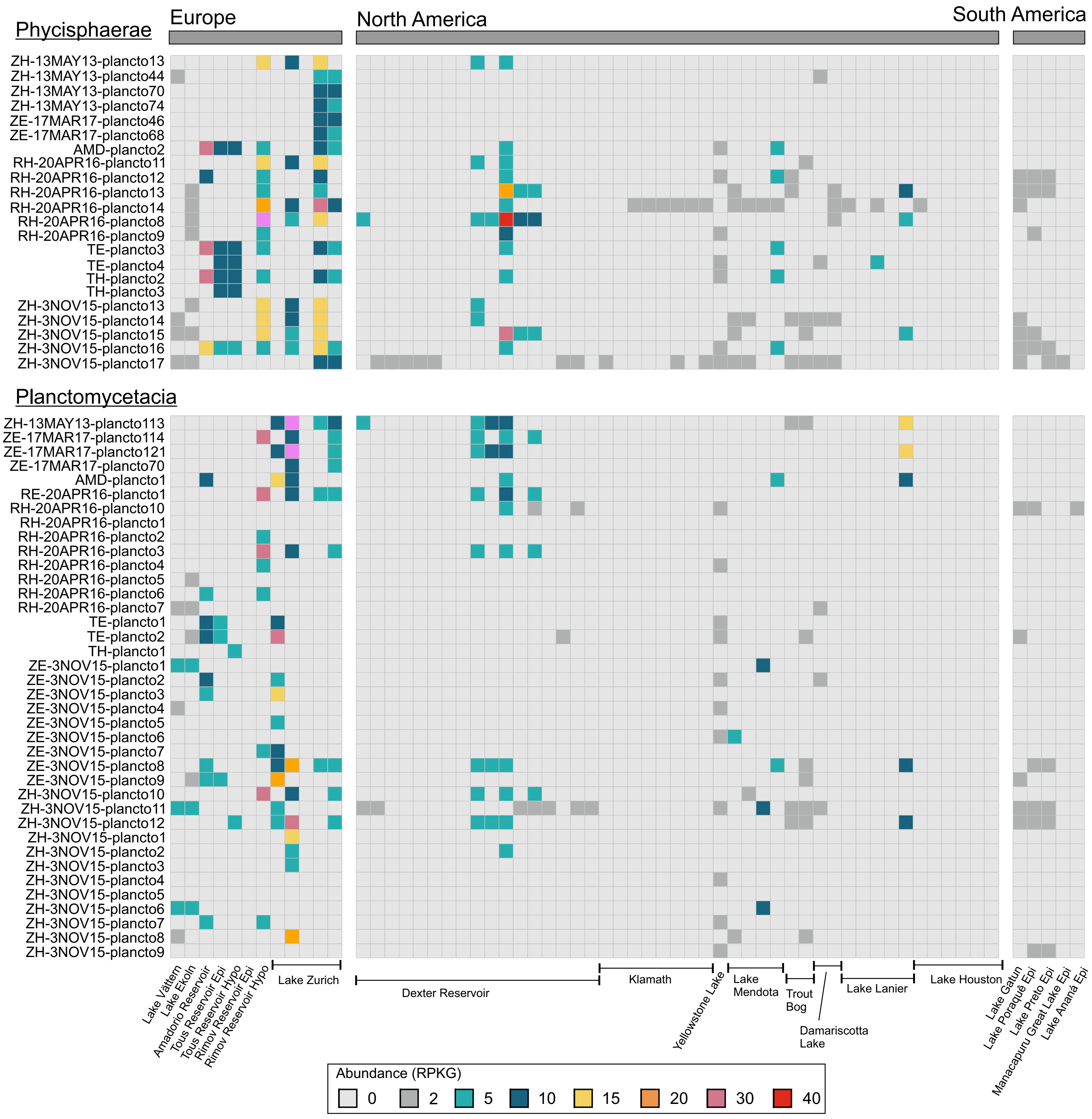

Supplementary Figure 8. Heat-map of MAGs abundance (expressed as RPKG values) in 64 lacustrine datasets. The left side of the heat-map shows the MAGs grouped by phylogeny, while the bottom part of the figure shows the geographic distribution of the sample collection sites.

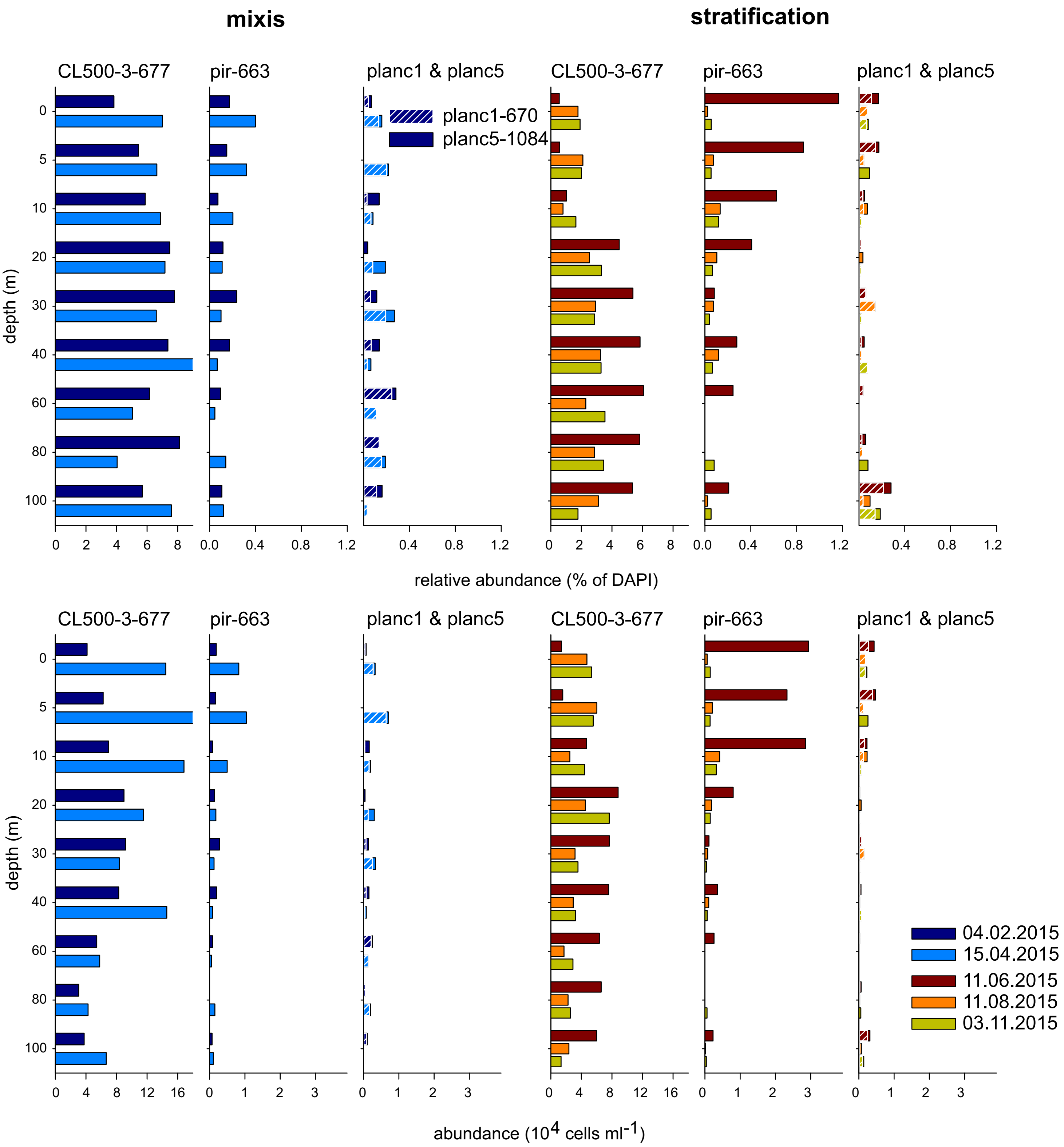

Supplementary Figure 9. Vertical profiles of CARD-FISH abundances (top: relative, bottom: absolute) of four lineages of Planctomycetes in Lake Zurich during two different sampling periods in 2015 (mixis and stratification).

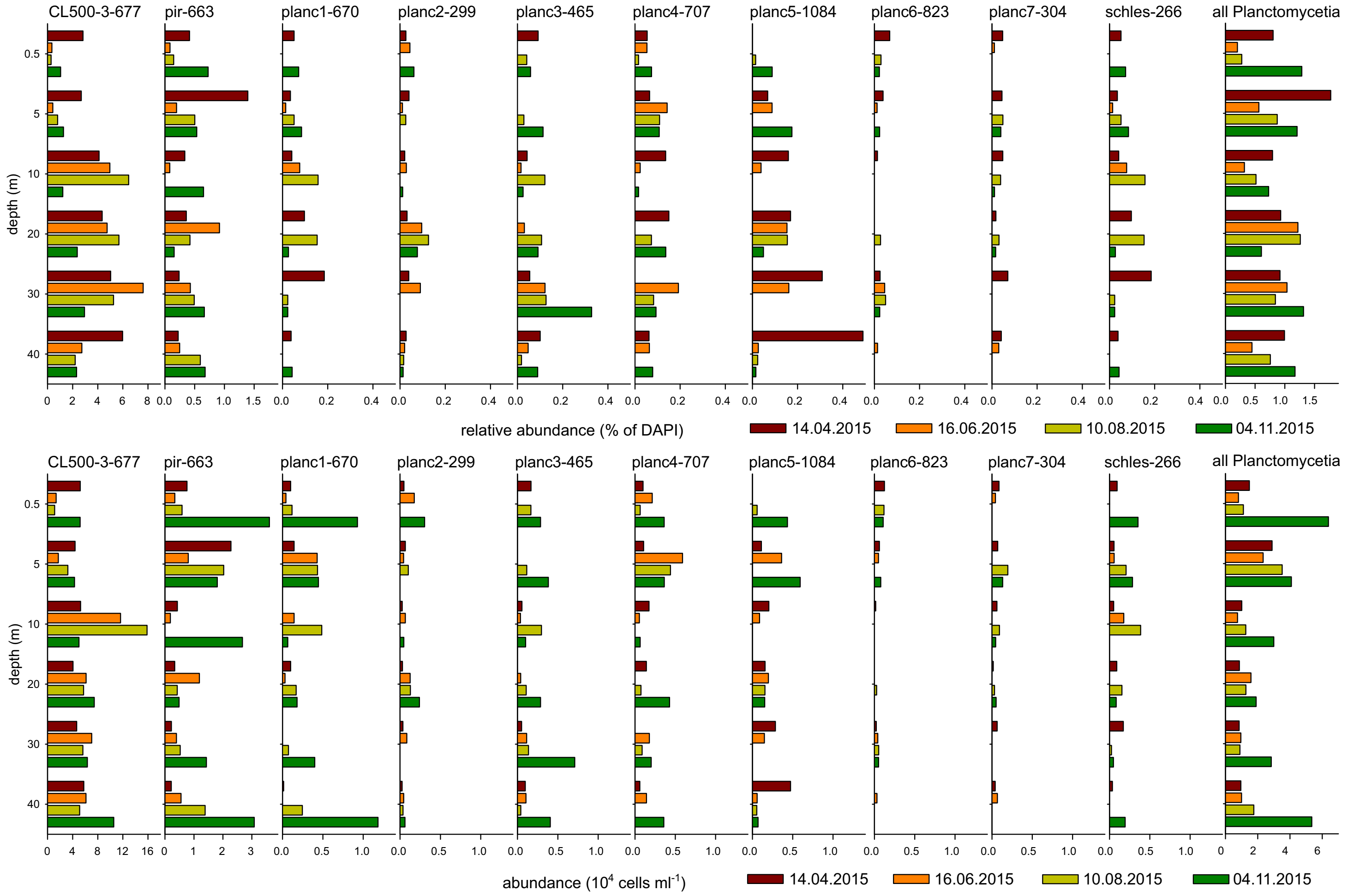

Supplementary Figure 10. Vertical profiles of CARD-FISH abundances (top:relative, bottom:absolute) of ten lineages of Planctomycetes in the Rimov reservoir during four different samplings in 2015.

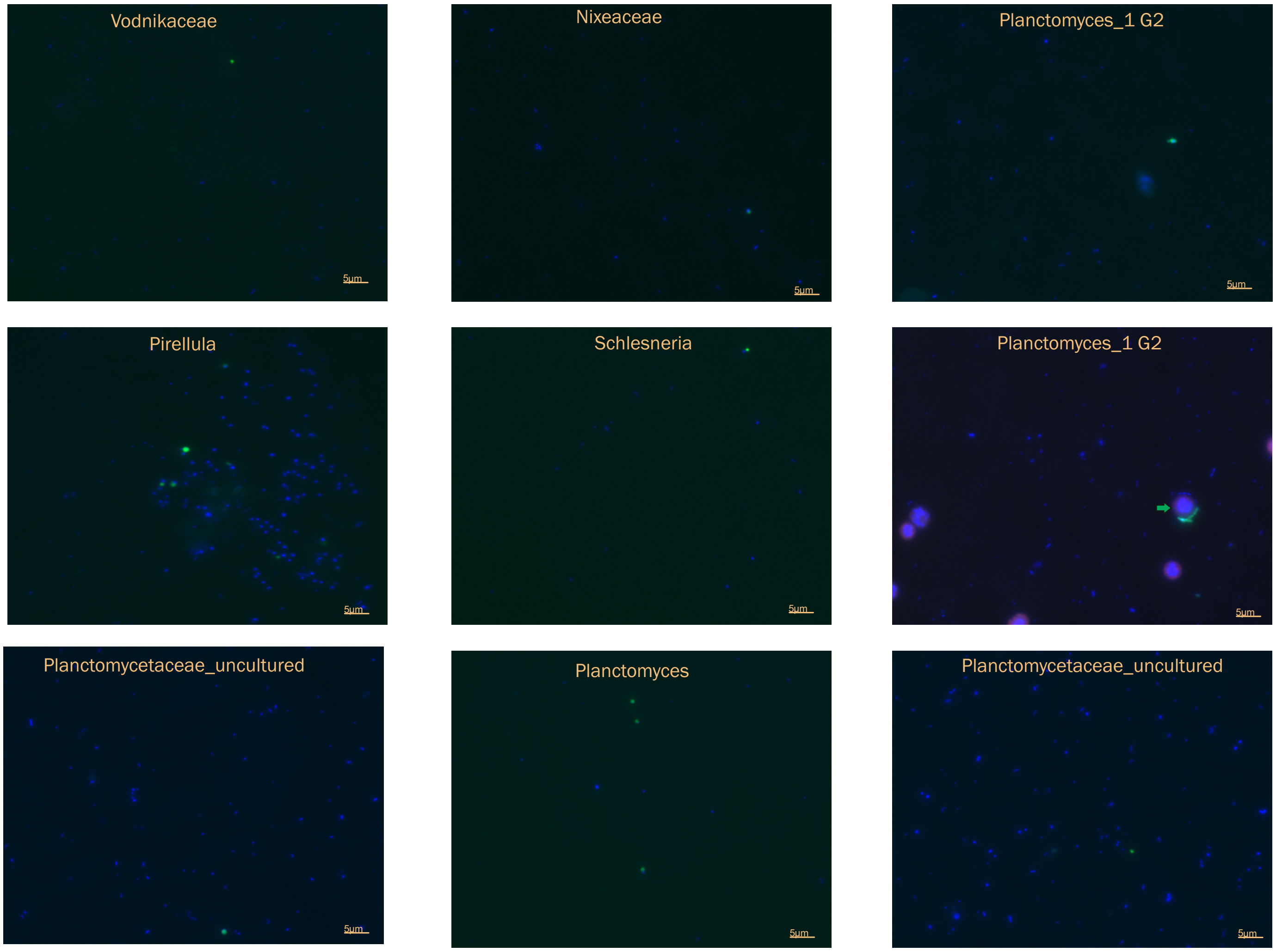

Supplementary Figure 11. Superimposed images of CARD-FISH stained Planctomycetes (green color) and DAPI-stained prokaryotes (blue color). Red flourescence is from Cyanobacteria (Microcystis, marked with a green arrow). The names of the panels are in accordance with Supplementary Figure 4. The scale bar is 5μm.

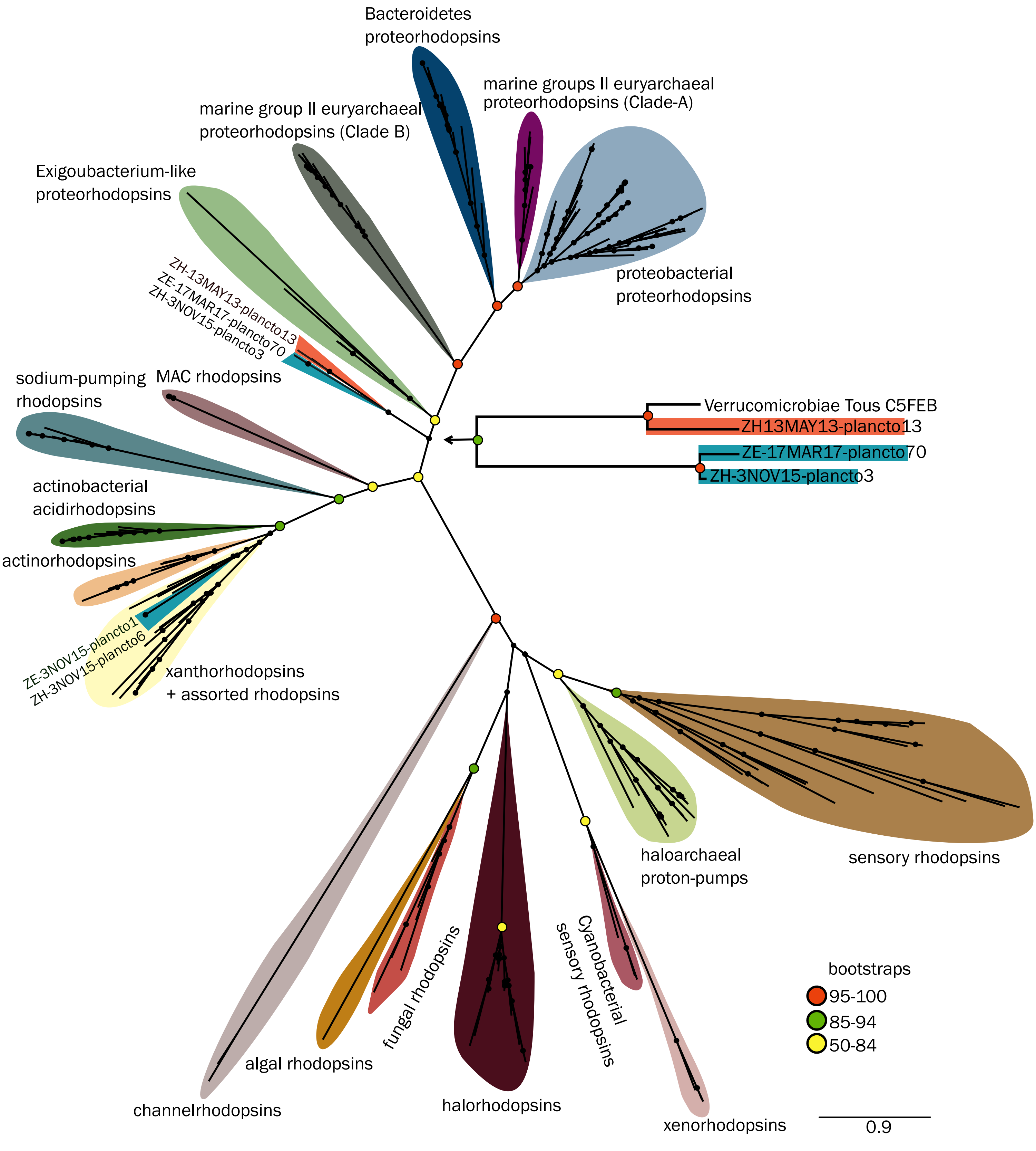

Supplementary Figure 12. Maximum-likelihood phylogenetic tree constructed using 254 rhodopsin sequences derived from diverse environments (freshwater, brackish, marine and hypersaline). The sequences belonging to freshwater MAGs are depicted in a subtree at the right side of the main figure

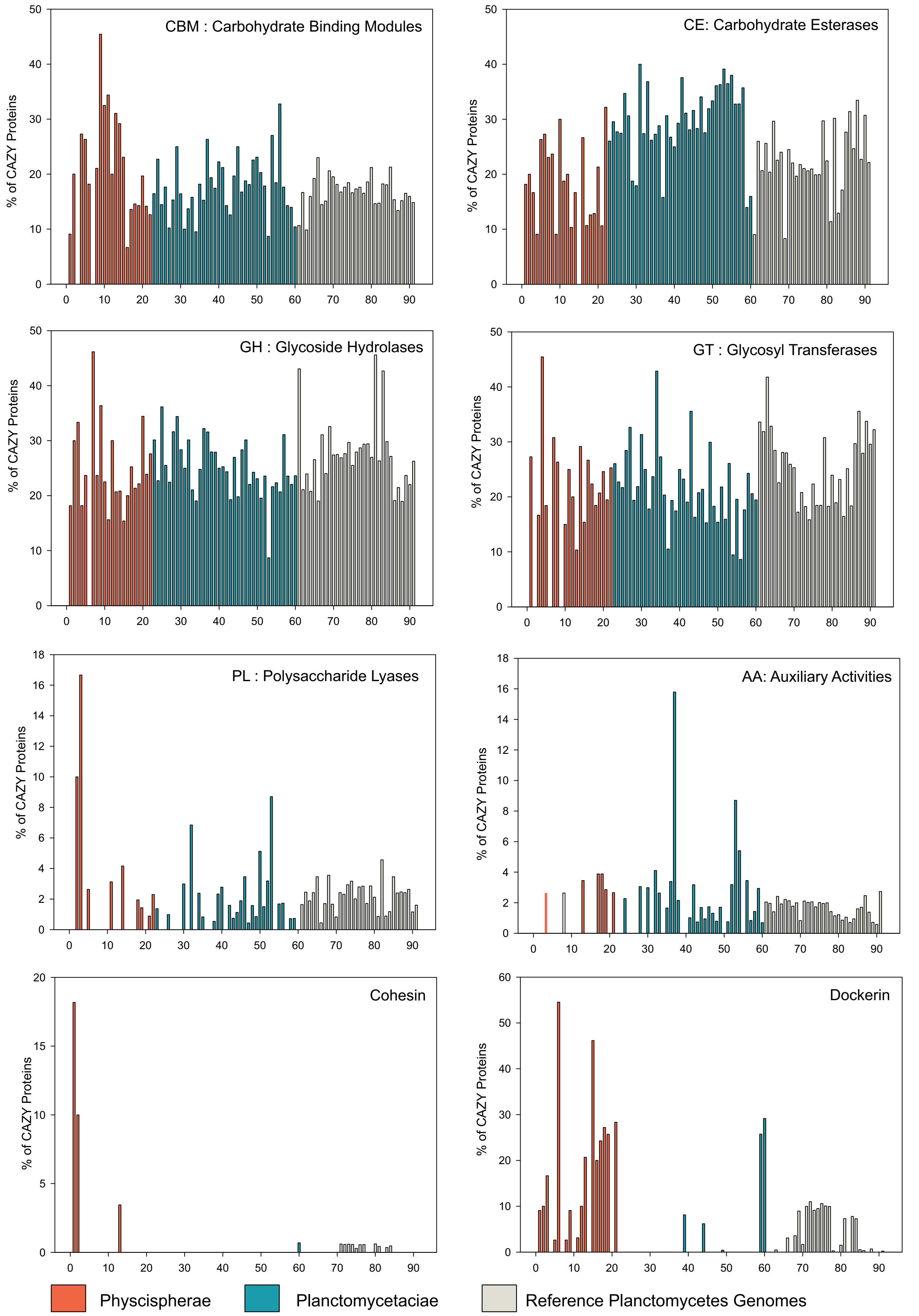

Supplementary Figure 13. Families of structurally-related catalytic and carbohydrate-binding modules (CAZy proteins) found in freshwater Planctomycetes MAGs and reference genomes.

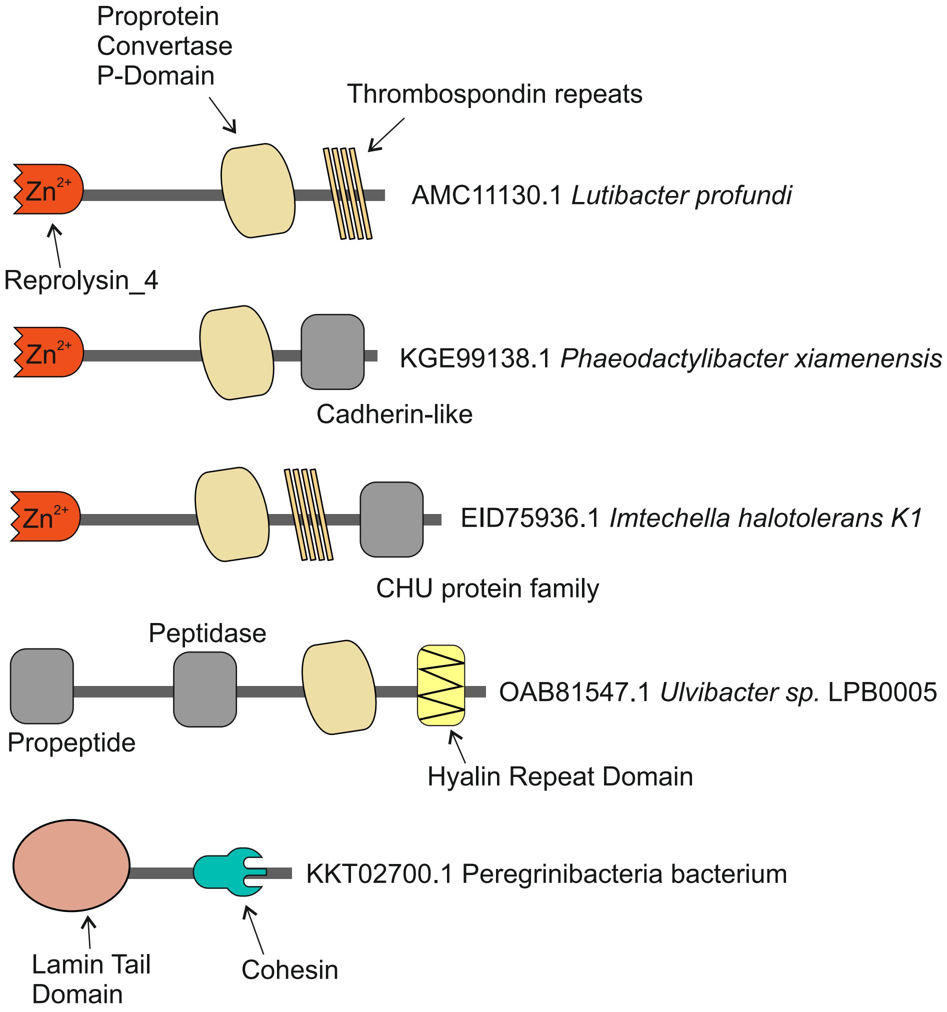

Supplementary Figure 14. Planctosome protein domains (shown in colors) found in proteins belonging to diverse bacterial species. Other domains are shown in grey.
